# Supplementary material for: O-Glycan-Dependent Interaction between MUC1 Glycopeptide and MY.1E12 Antibody by NMR, Molecular Dynamics and Docking Simulations
Source: Int J Mol Sci. 2022 Jul 16;23(14):7855. doi: 10.3390/ijms23147855 (PMC9322718; doi:10.3390/ijms23147855)
Supplement: Supplementary file 1 [file ijms-23-07855-s001.zip › Suppementary Tables.pdf]

Table S1. Chemical shift of MUC1 9AA (278K, referenced to DSS)

Residue	NH	αH	βH	Others
A4 (Ac)	8.43	4.20	1.28	-
H5	8.52	4.66	3.13<br>3.24	δH 8.16<br>εH 7.14
G6	8.47	3.94	-	-
V7	8.20	4.31	2.09	γH 1.00
T8	9.00	4.68	4.34	γH 1.25
S9	8.65*	4.47*	3.75<br>3.84	-
A10	8.65*	4.47*	1.39	-
P11	-	4.42	2.30<br>2.04	γH 2.06, 2.06<br>δH 3.64, 3.82
D12	8.10	4.32	2.52	-
GalNAc	7.63	-	-	H2 4.20 H3 4.03 H4 3.76
Gal	-	-	-	H1 4.96<br>H2-H5 ND
NeuAc	8.17	-	-	H3(ax) 1.78<br>H3(eq) 2.74<br>H4 3.67<br>H5 3.83

ND: not determined. *: Overlapping

Table S2. Chemical shift of MUC1 20AA (278K, referenced to DSS)

Residue	NH	αH	βH	Others
A1 (Ac)	8.59	4.26	1.33	-
P2	-	4.40	2.30<br>2.30	γH ND<br>δH ND
P3	-	ND	ND<br>ND	γH ND<br>δH ND
A4	8.22*	4.31*	1.20	-
H5	8.41	4.60	3.16<br>3.16	δH 8.05<br>εH 7.10
G6	8.49	4.02	-	-
V7	8.22*	4.31*	2.11	γH 0.98, ND
T8	9.02	4.68	4.35	γH 1.27
S9	8.65*	4.46*	3.76<br>3.85	-
A10	8.65	4.56	1.37	-
P11	-	ND	ND<br>ND	γH ND<br>δH ND
D12	8.64	4.61	2.76<br>2.65	-
T13	8.22*	4.31*	0.98	0.97
R14	8.41	4.61	1.78<br>1.84	γH 1.71, 1.71<br>δH 3.14, 3.14<br>NH 7.44
P15	-	ND	ND<br>ND	γH ND<br>δH ND
A16	8.65*	4.46*	1.37*	-
P17	-	ND	ND<br>ND	γH ND<br>δH ND
G18	8.72	4.01	-	-
S19	8.29	4.56	3.92<br>3.94	-
T20	7.96	4.19	1.18	γH 1.19
GalNAc	7.63	-	-	ND
Gal	-	-	-	H1 4.95<br>H2-H5 ND
NeuAc	8.18	-	-	H3(ax) 1.77<br>H3(eq) 2.76<br>H5 3.84

ND: not determined. *: Overlapping

Table S3. Chemical shift of MUC1 27AA (278K, referenced to DSS)

Residue	NH	αH	βH	Others
A1	ND	ND	ND	-
P2	-	ND	ND<br>ND	γH ND<br>δH ND
P3	-	ND	ND	γH ND<br>δH ND
A4	ND	ND	ND	-
H5	ND	ND	ND<br>ND	δH 7.10<br>εH 8.05
G6	8.51*	3.85*	-	-
V7	8.23	4.30	2.09	γH 0.98, 0.98
T8	9.04	4.68	ND	γH 1.26
S9	ND	ND	ND<br>ND	-
A10	ND	ND	ND	-
P11	-	ND	ND<br>ND	γH ND<br>δH ND
D12	8.64	4.61	2.66<br>2.75	-
T13	8.23	4.30	4.32	γH 0.98
R14	8.41	4.60	1.83<br>ND	γH 1.72<br>δH 3.20<br>NH 7.46
P15	-	ND	ND<br>ND	γH ND<br>δH ND
A16	ND	ND	ND	-
P17	-	ND	ND<br>ND	γH ND<br>δH ND
G18	8.71	3.98	-	-
S19	8.29	4.52	3.89<br>3.93	-
T20	8.39	4.37	4.22	γH 1.21
A21	8.46	4.59	1.35	-
P22	-	ND	ND	γH ND<br>δH ND
P23	-	ND	ND	γH ND<br>δH ND
A24	ND	ND	ND	-
H25	ND	ND	ND<br>ND	δH 7.14<br>εH 8.16
G26	8.51*	3.85*	-	-
V27	7.79	4.09	2.13	γH 0.91, 0.88
GalNAc	7.64	-	-	H2-H4 4.20
Gal	-	-	-	H1 4.96<br>H2-H5 ND

NeuAc	8.19	-	-	H3(ax) 1.78<br>H3(eq) 2.75<br>H4 3.67<br>H5 3.85<br>H6 3.61

ND: not determined. *: Overlapping
