# Supplementary material for: O-Glycan-Dependent Interaction between MUC1 Glycopeptide and MY.1E12 Antibody by NMR, Molecular Dynamics and Docking Simulations
Source: Int J Mol Sci. 2022 Jul 16;23(14):7855. doi: 10.3390/ijms23147855 (PMC9322718; doi:10.3390/ijms23147855)
Supplement: Supplementary file 1 [file ijms-23-07855-s001.zip › Supplementary Figures.pdf]

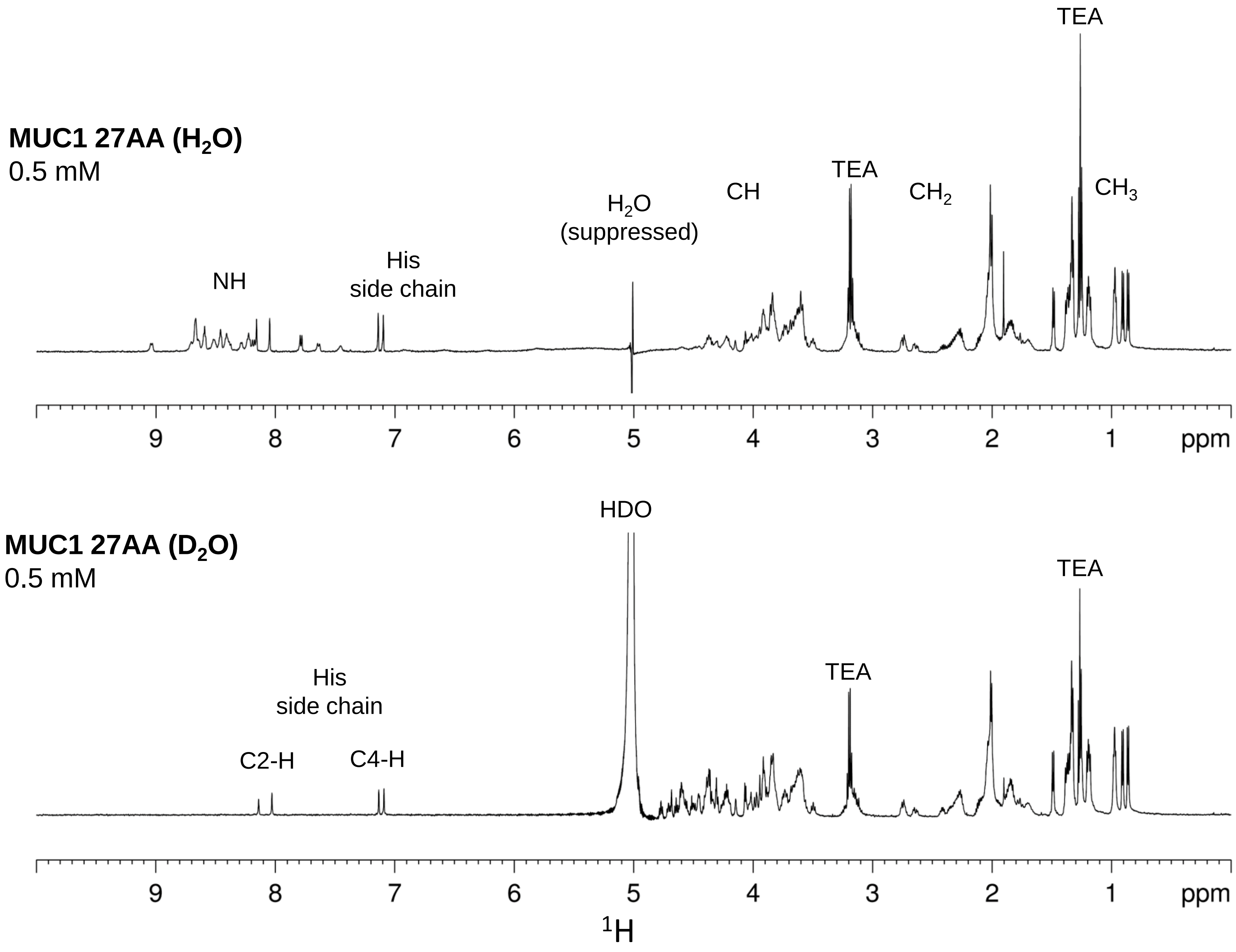

Figure S1. 600 MHz ^{1}H-NMR spectra of MUC1 glycopeptide (27AA) in H_2O or D_2O at 278 K.

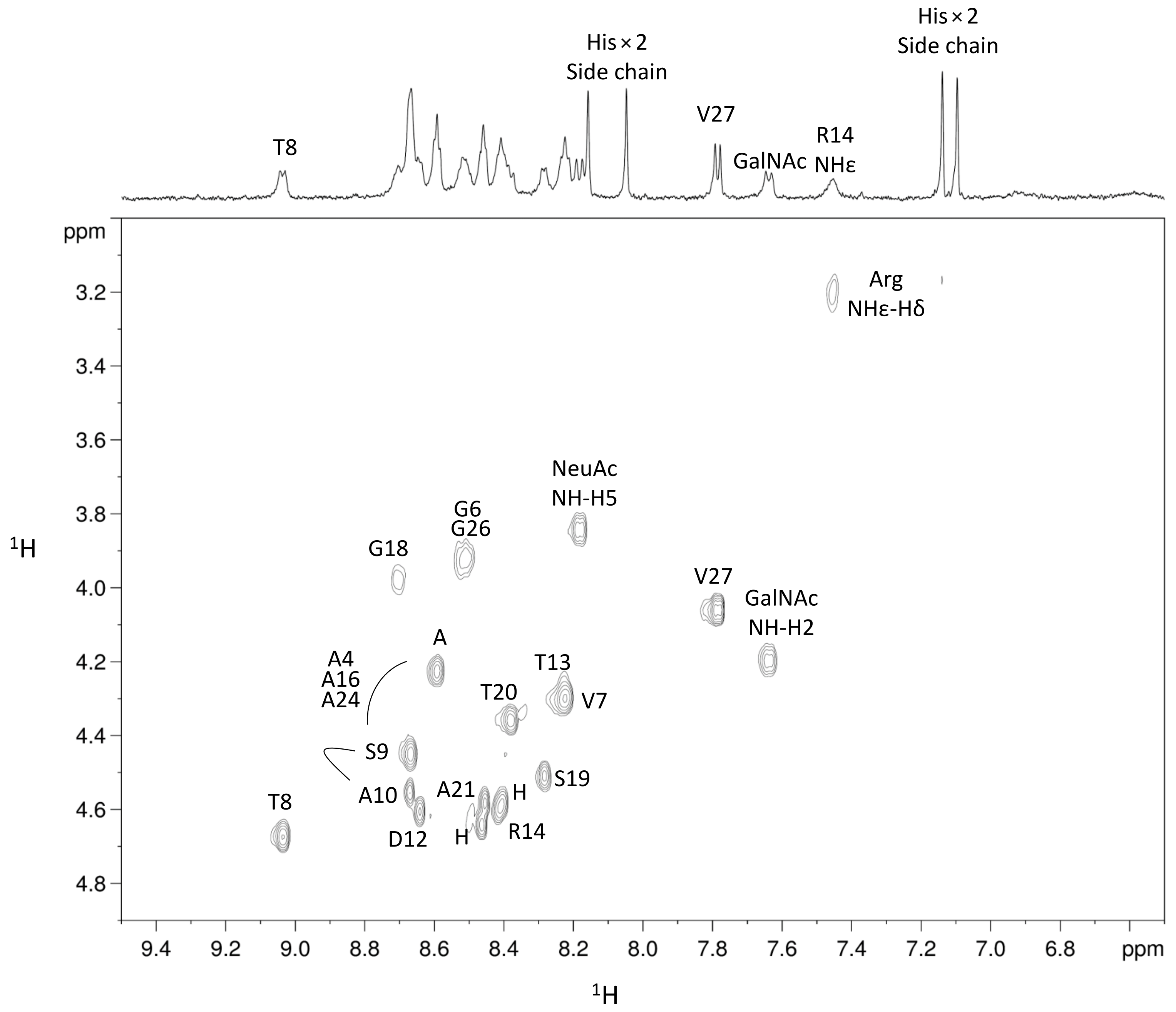

Figure S2. 2D CLIP-COSY spectrum of 0.5 mM MUC1 glycopeptide (27AA) (NH-Hα region) at 278 K.

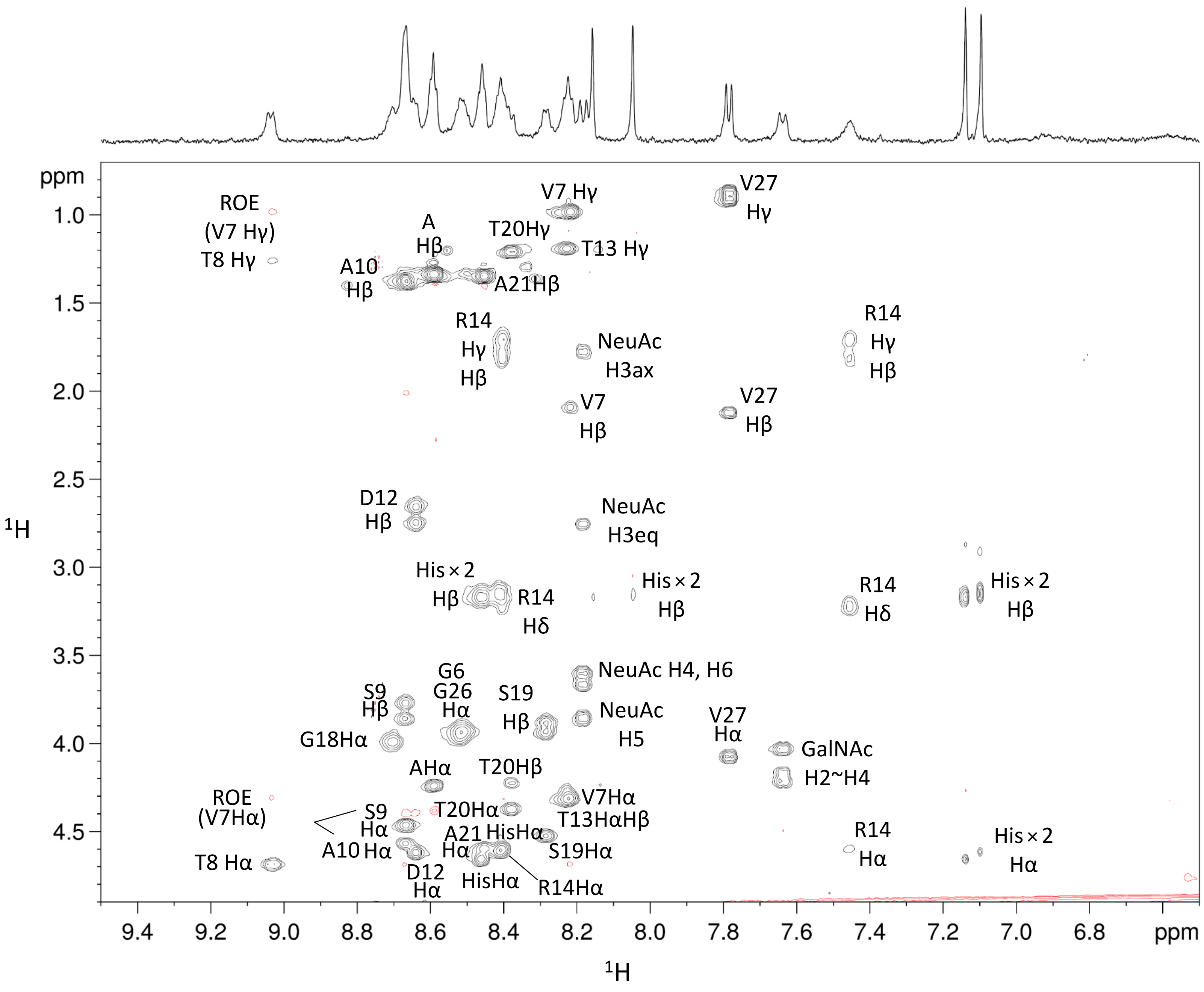

Figure S3. 2D TOCSY spectrum of 0.5 mM MUC1 glycopeptide (27AA) (NH-aliphatic region) at 278K.

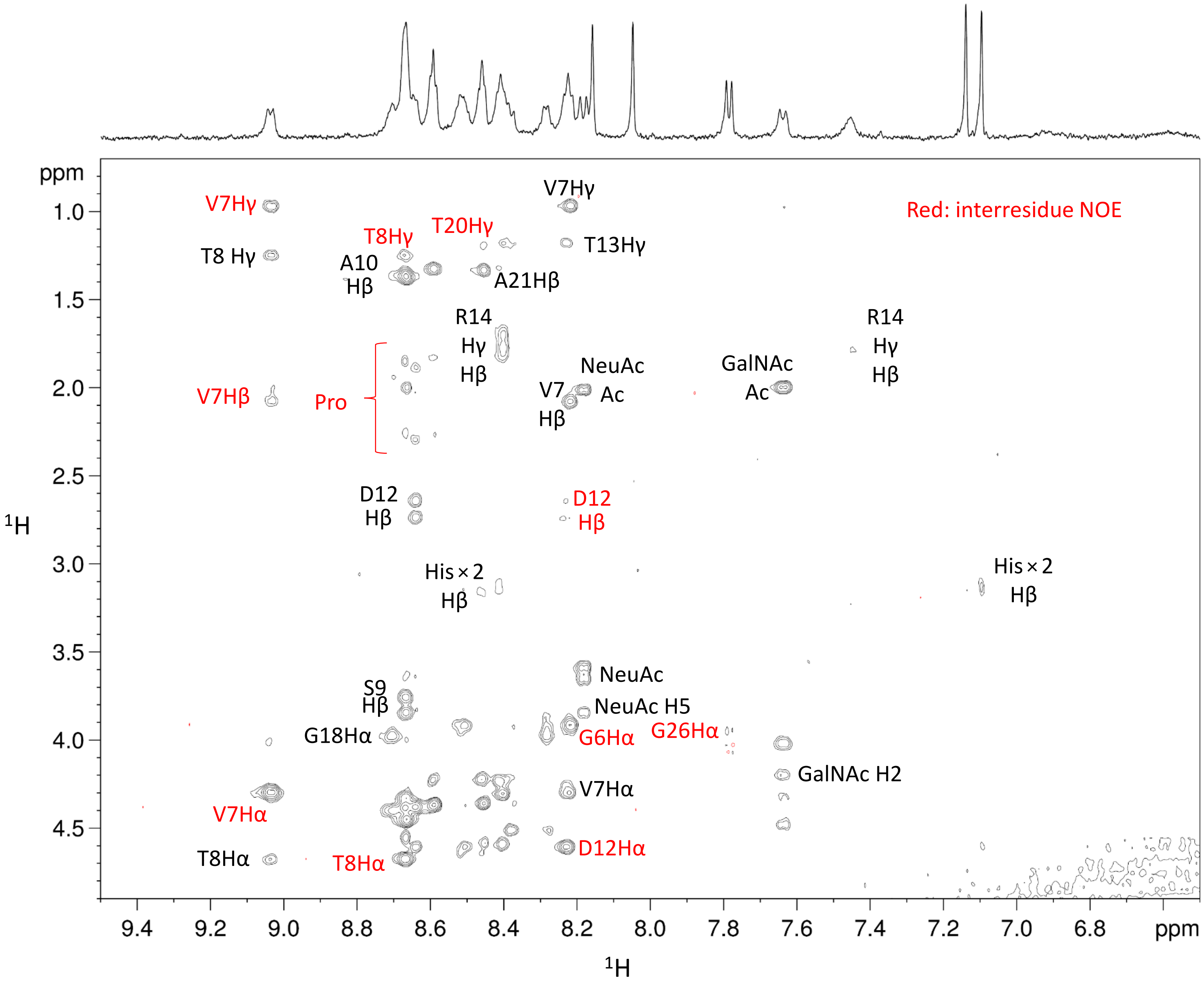

Figure S4. 2D NOESY spectrum of MUC1 glycopeptide (27AA) (NH-aliphatic region) at 278K.

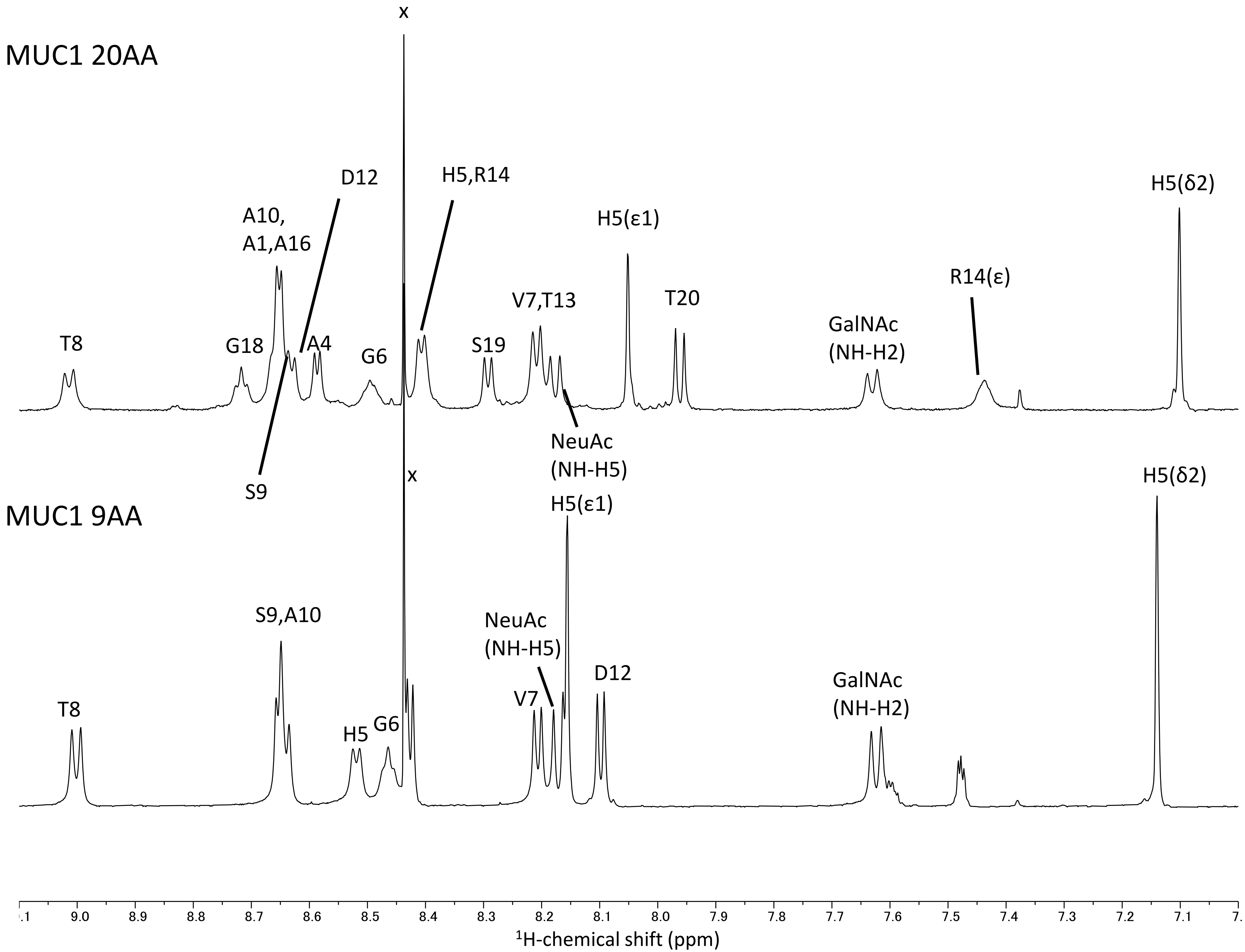

Figure S5. 1D-^{1}H NMR spectra (NH region) of MUC1 20AA (upper) and 9AA (lower). x: impurity.

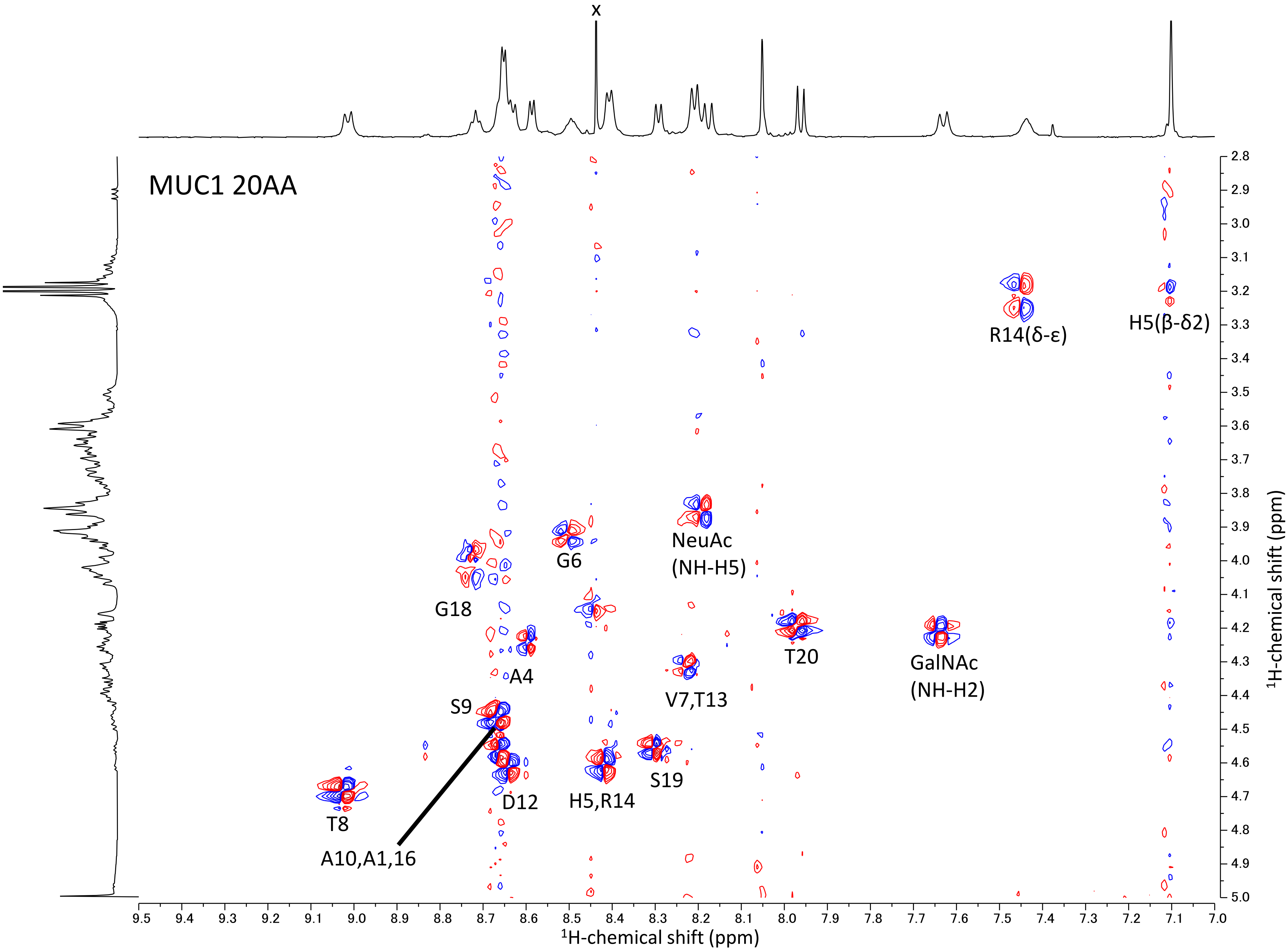

Figure S6. 2D DQF-COSY spectrum (NH-Hα region) of MUC1 (20AA). x: impurity.

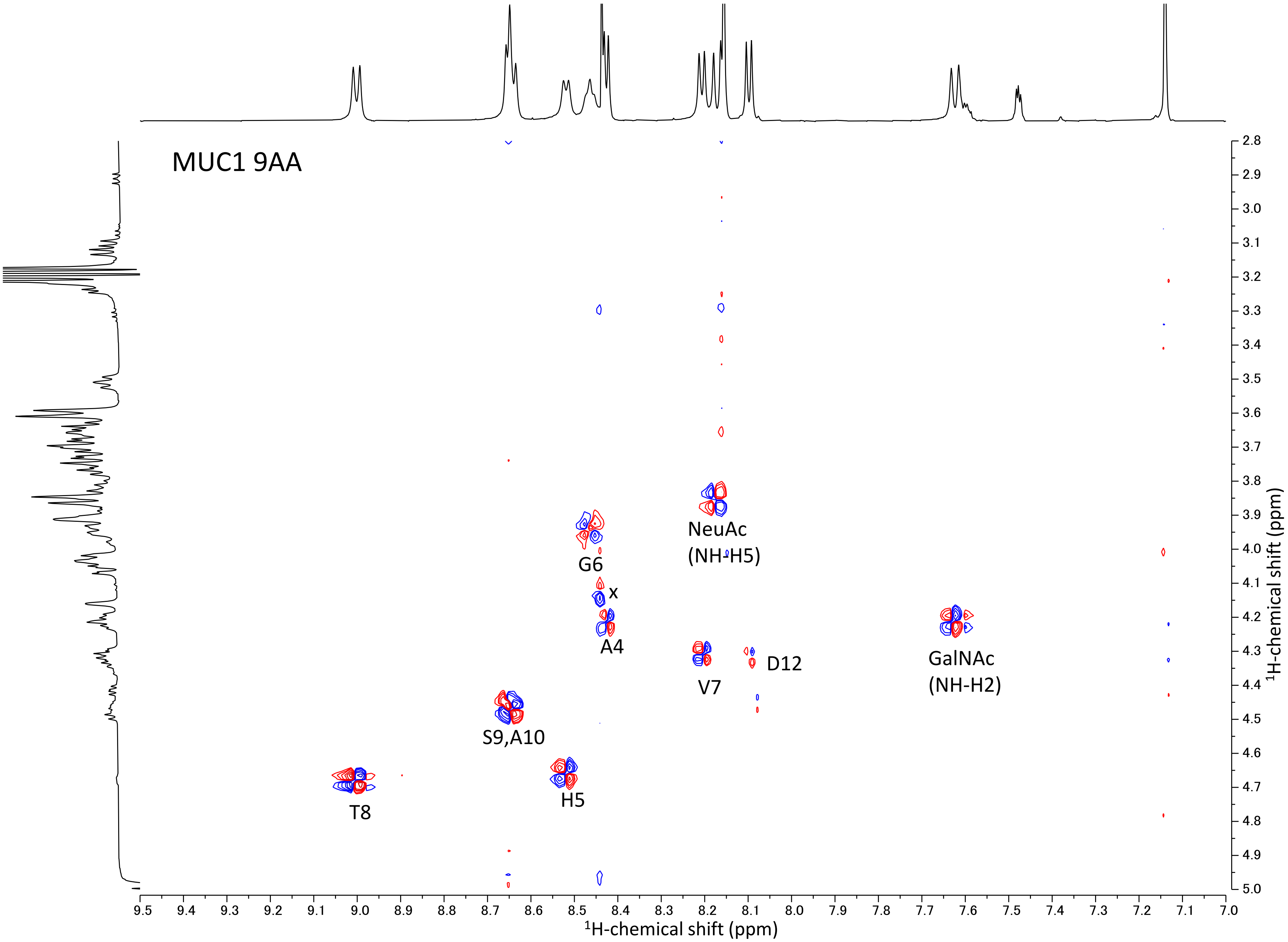

Figure S7. 2D DQF-COSY spectrum (NH-Hα region) of MUC1 (9AA). x: impurity.

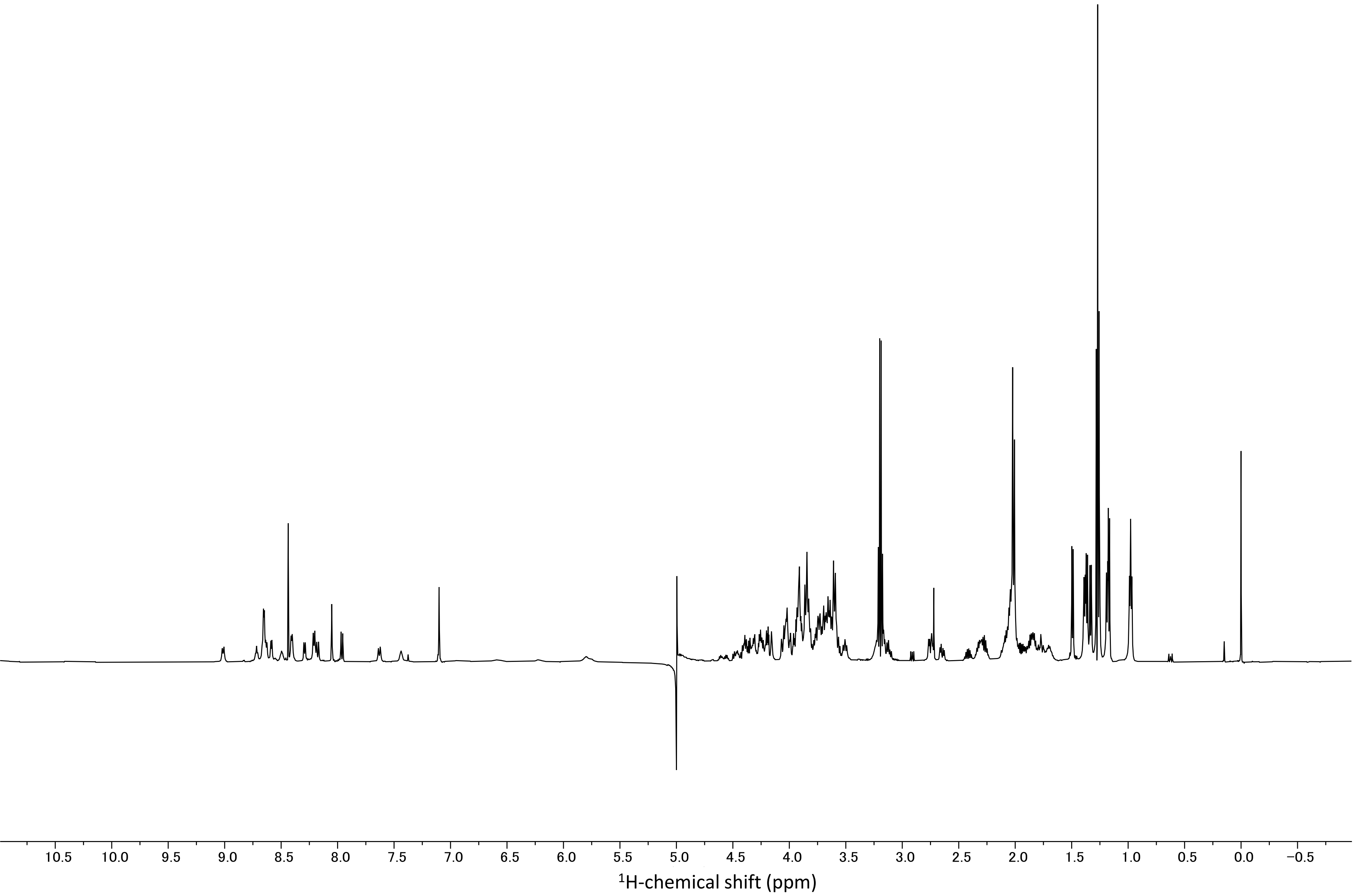

Figure S8. 1D-^{1}H NMR spectrum (full region) of MUC1 20AA.

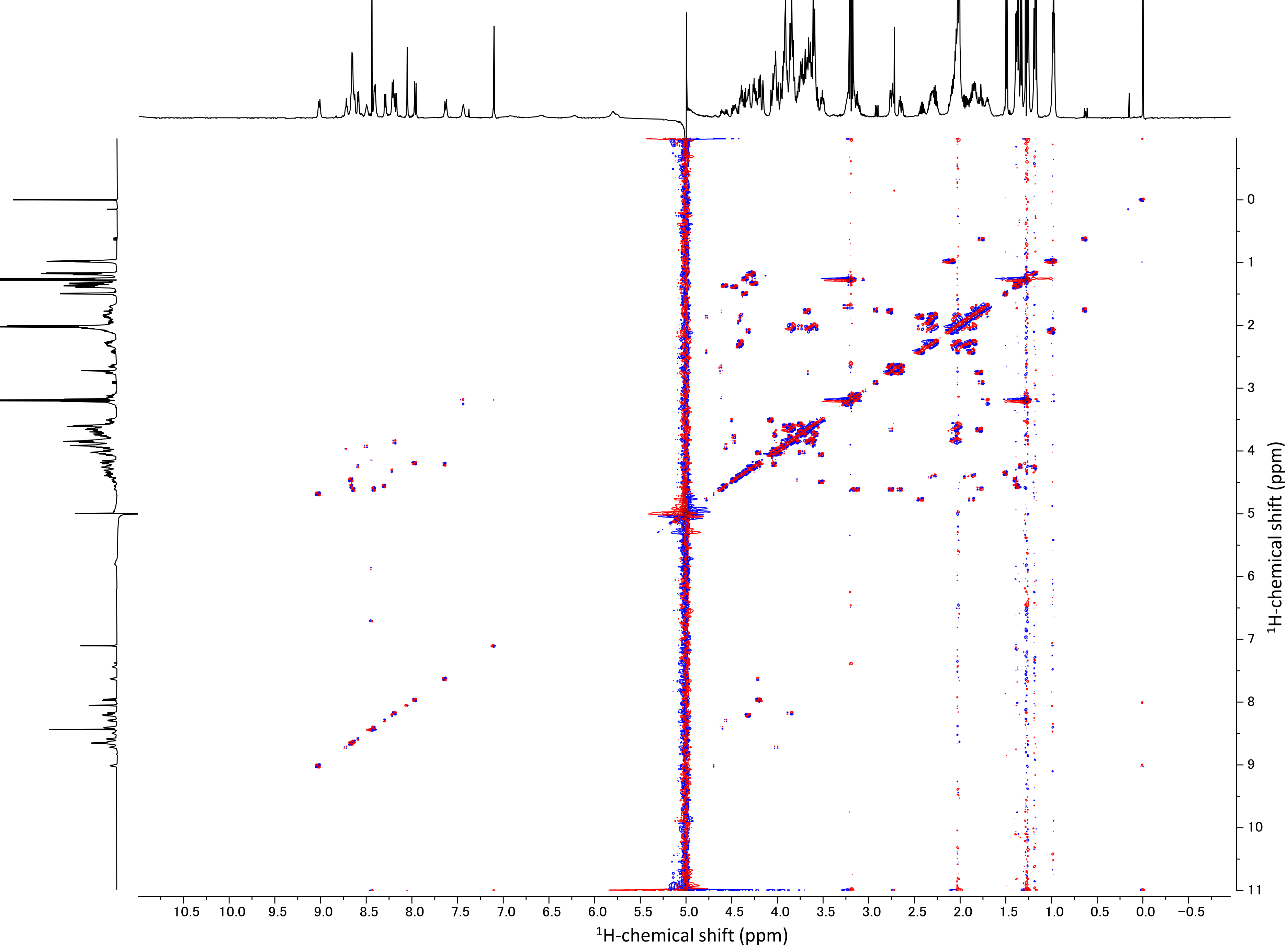

Figure S9. 2D DQF-COSY spectrum (full region) of MUC1 (20AA).

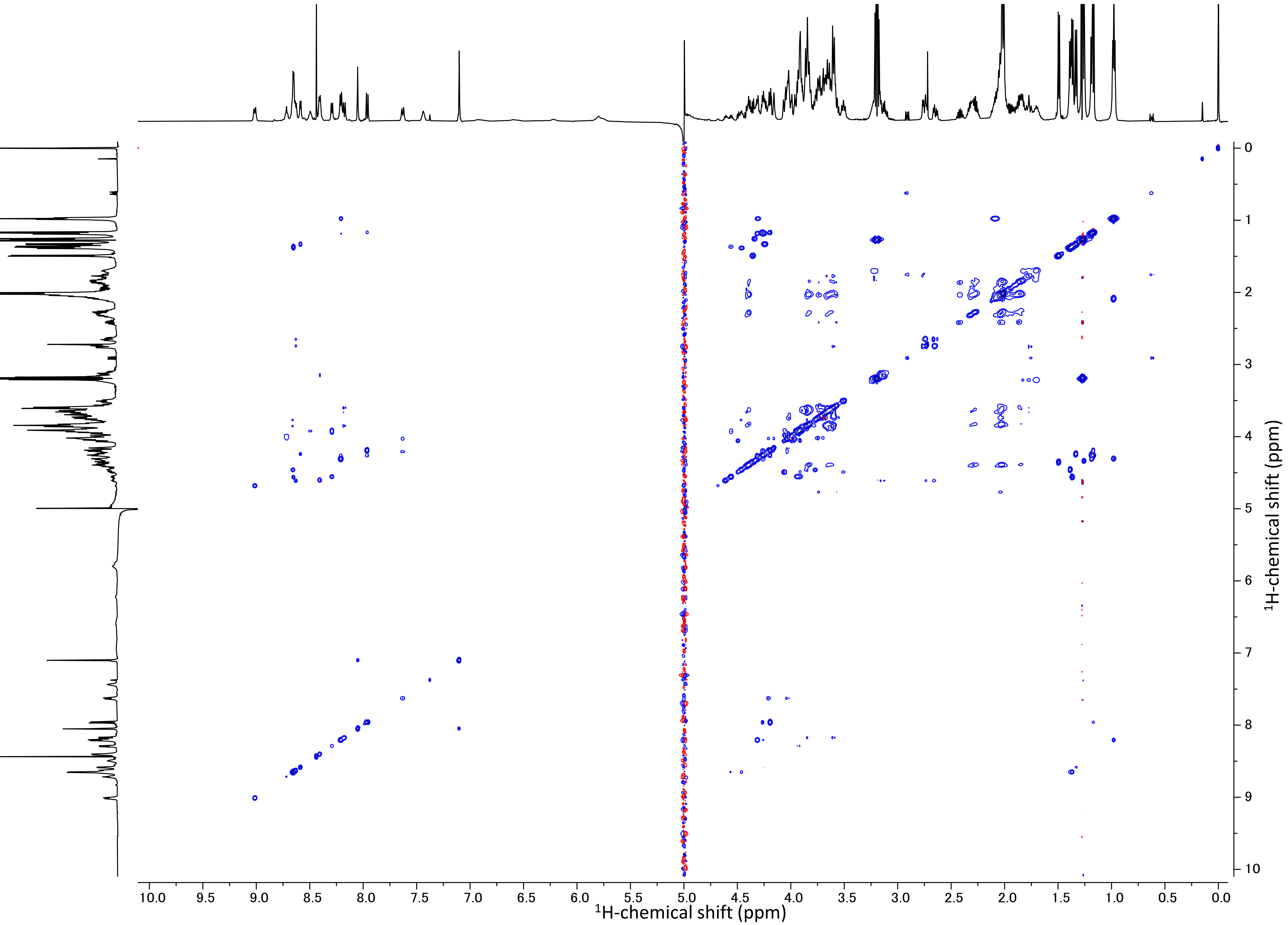

Figure S10. 2D HOHAHA spectrum (full region) of MUC1 (20AA).

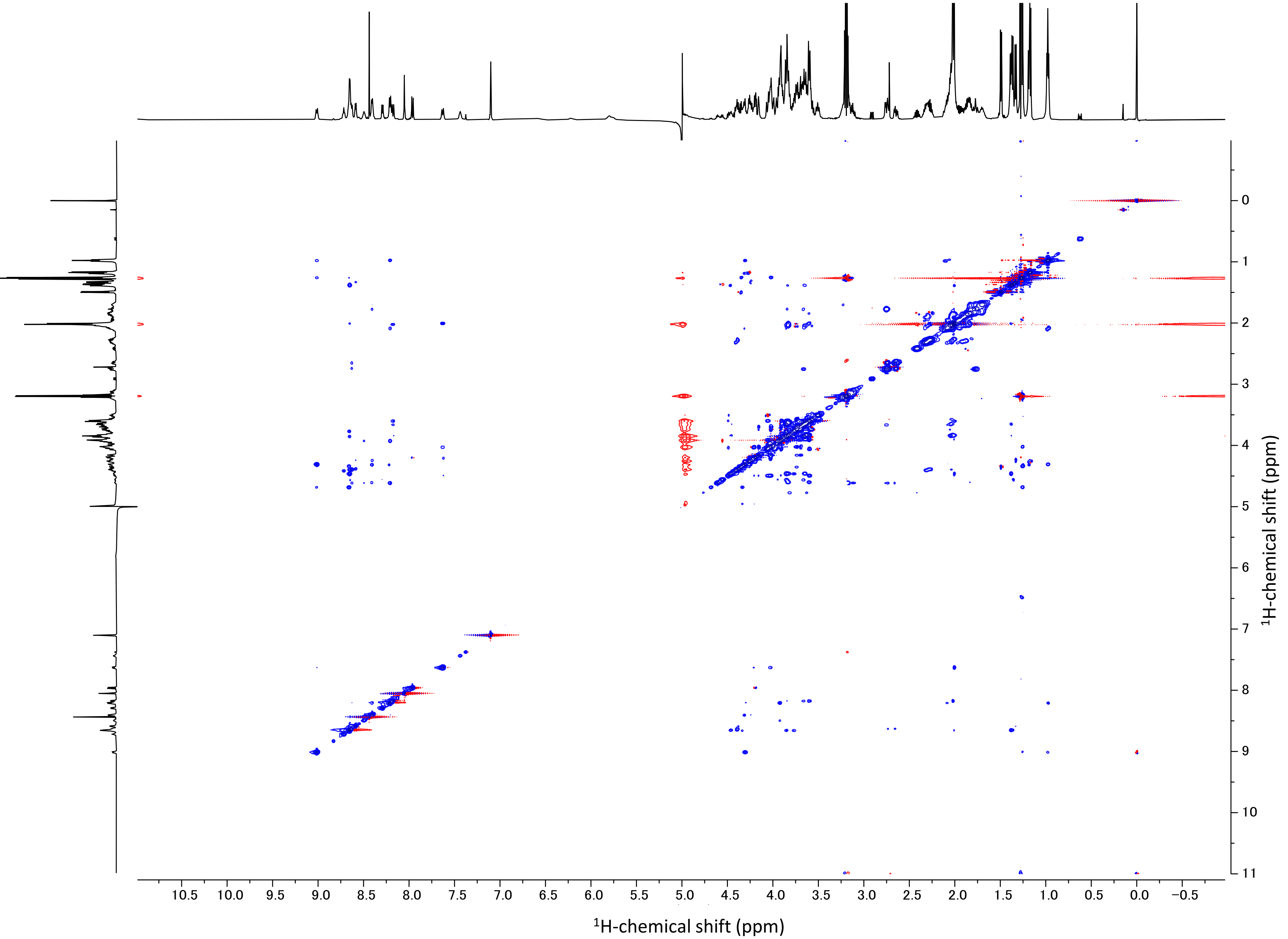

Figure S11. 2D NOESY spectrum (full region) of MUC1 (20AA).

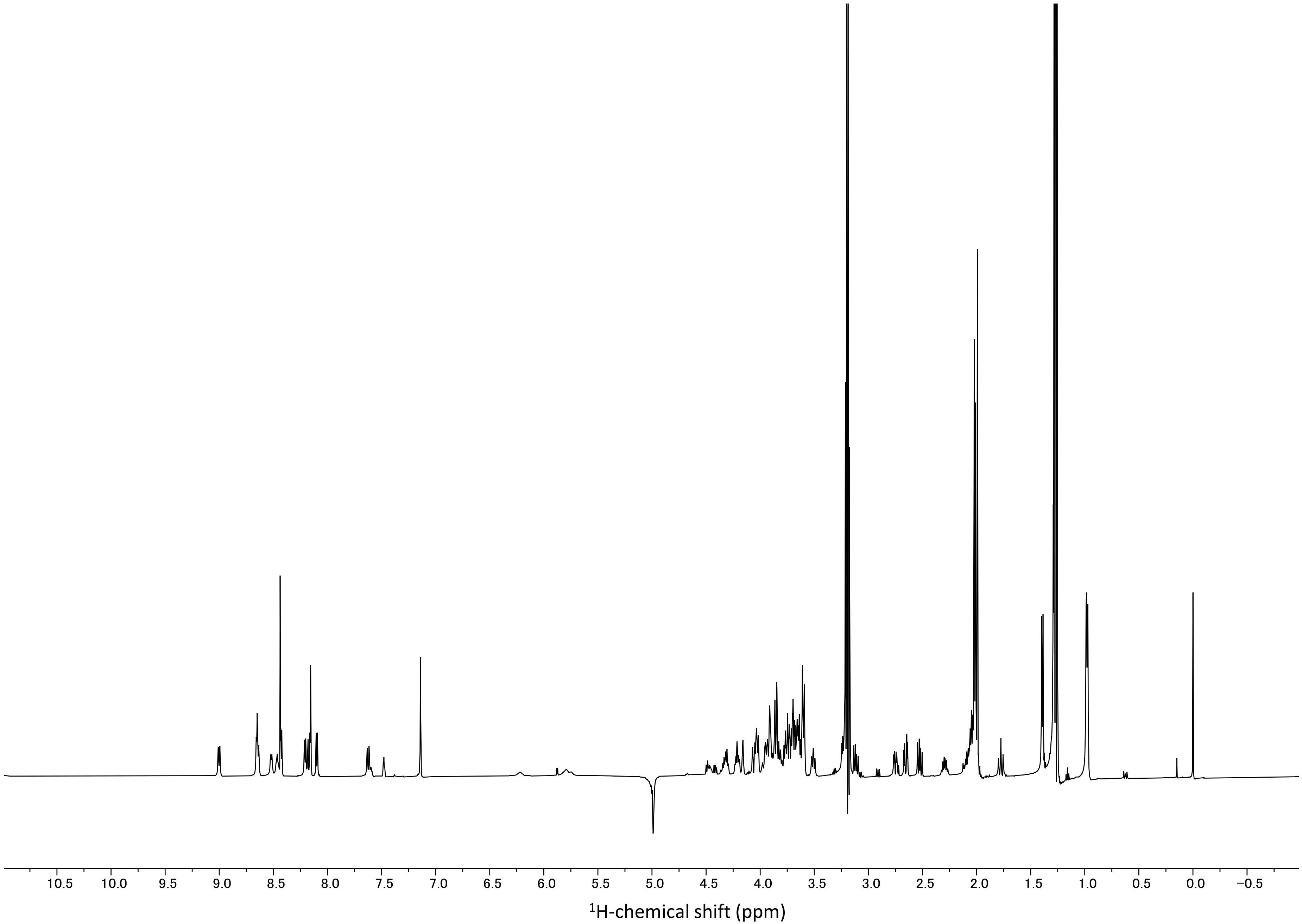

Figure S12. 1D-^{1}H NMR spectrum (all region) of 9AA.

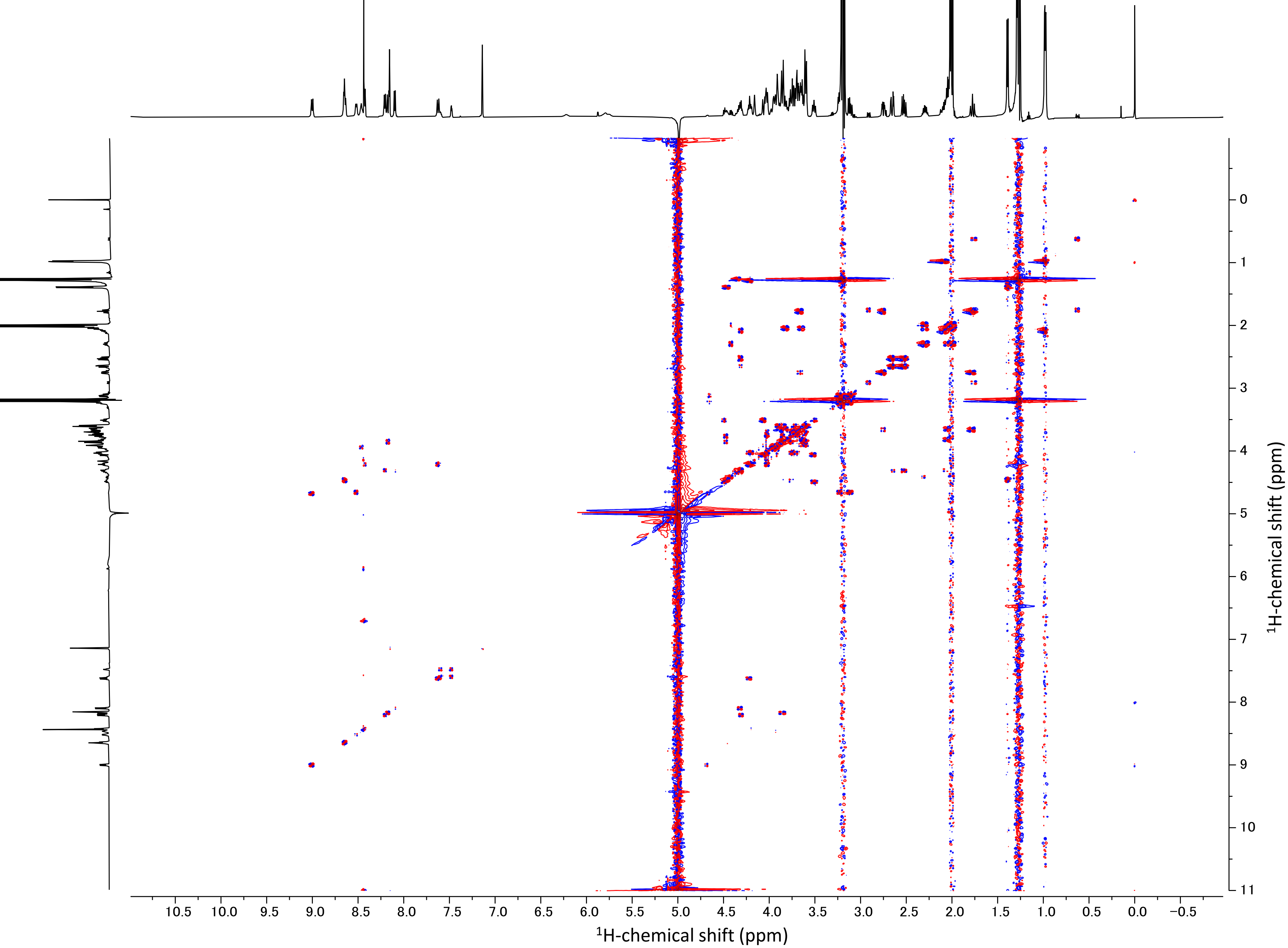

Figure S13. 2D DQF-COSY spectrum (full region) of MUC1 (9AA).

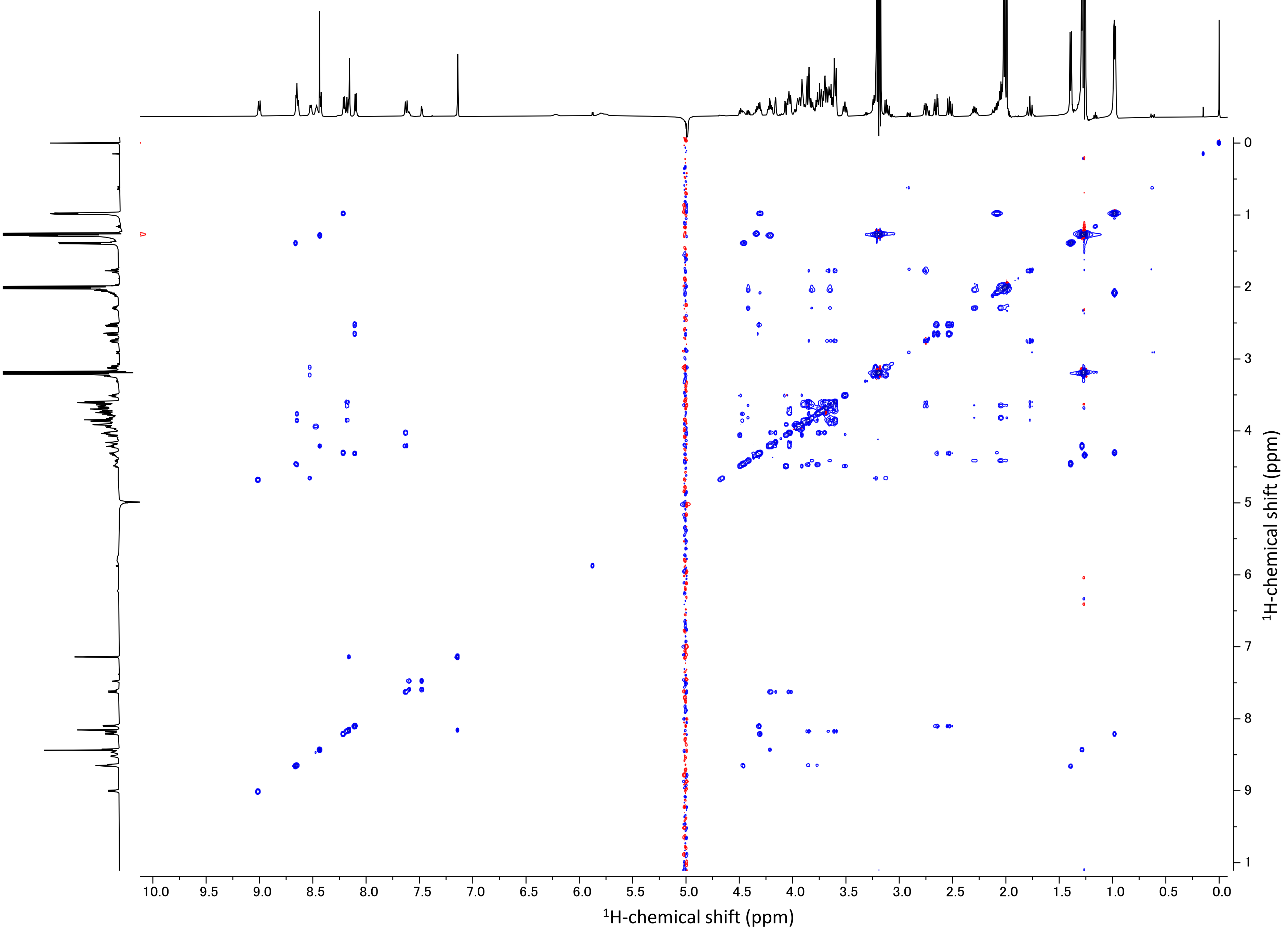

Figure S14. 2D HOHAHA spectrum (full region) of MUC1 (9AA).

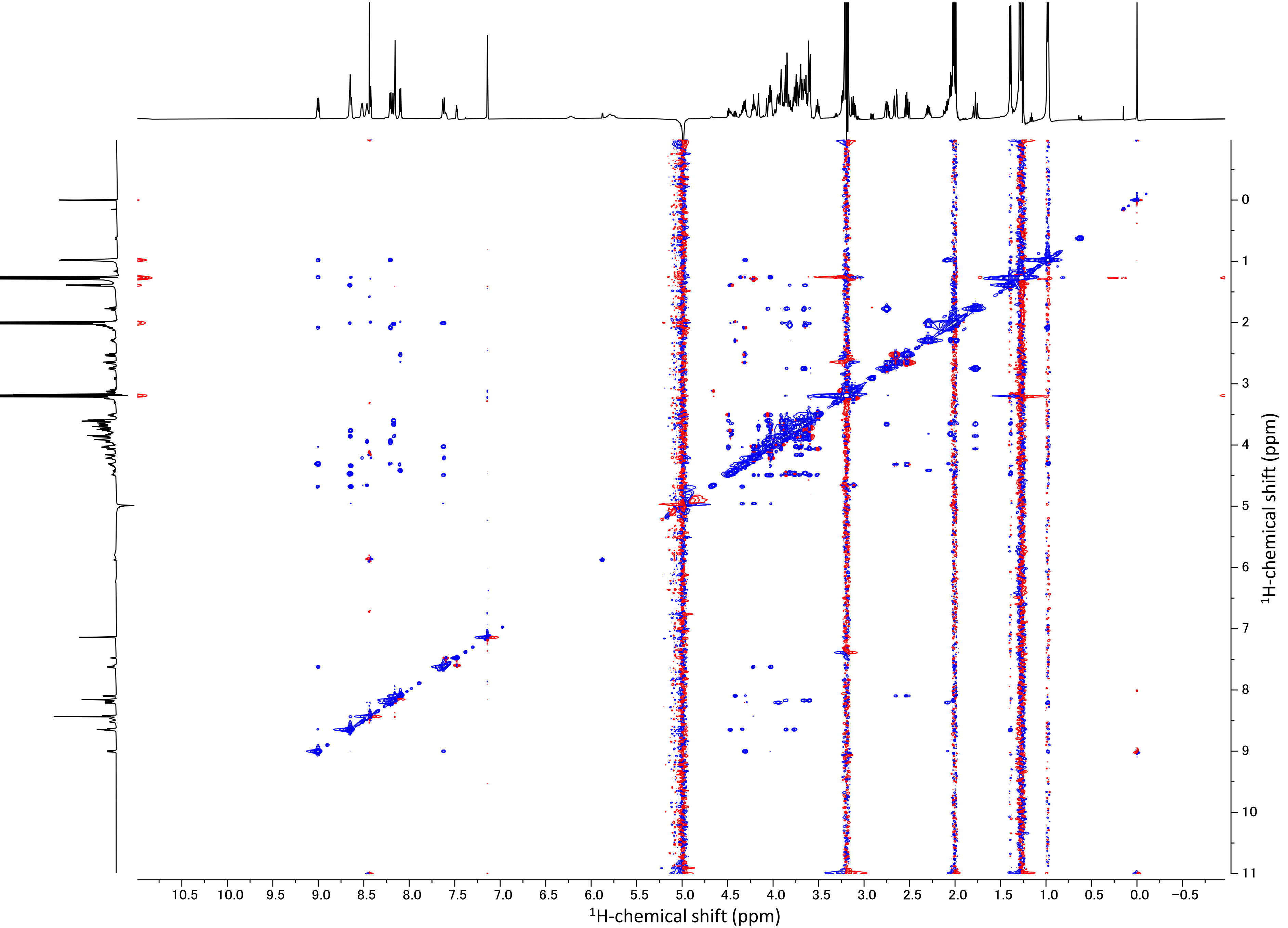

Figure S15. 2D NOESY spectrum (full region) of MUC1 (9AA).

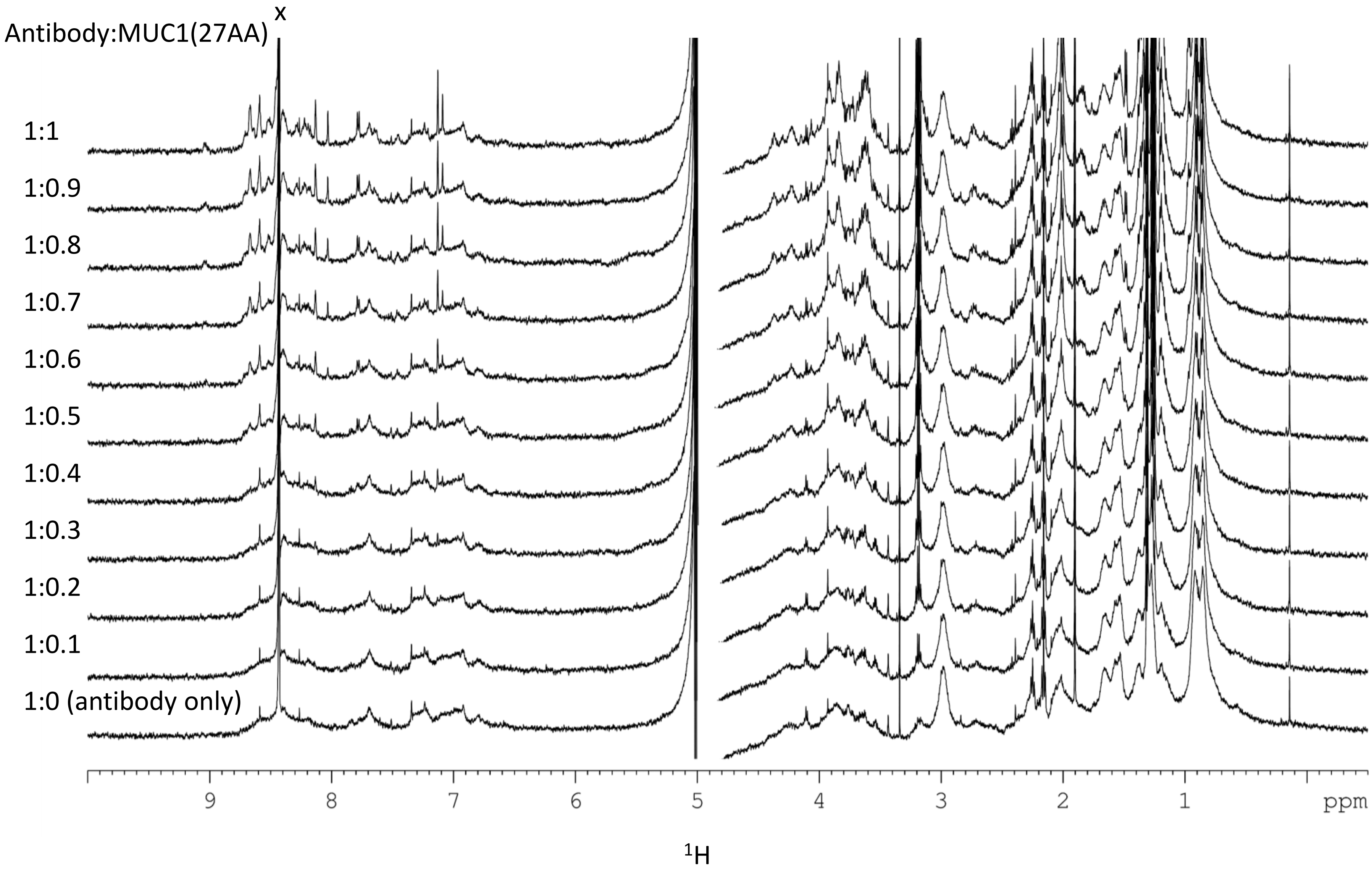

Figure S16: ^{1}H-NMR-monitored titration study of 25 μM MUC1 (27AA) in H_2O (whole spectra). Antibody(binding site):MUC1 molar ratio is indicated at the left.

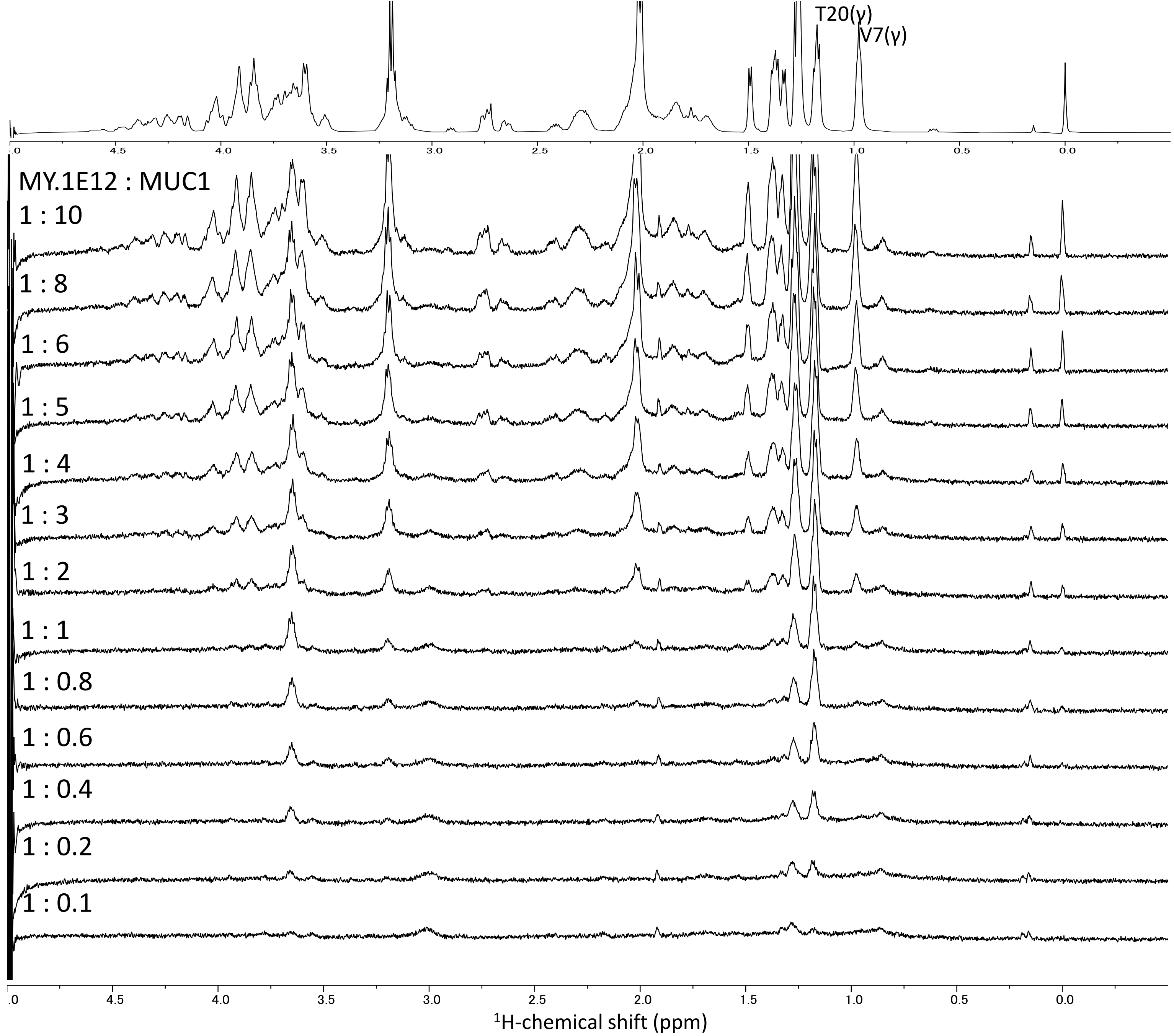

Figure S17. NMR titration experiments using MUC1 (20AA) and MY.1E12. 1D-^{1}H NMR spectra (aliphatic region) of MY.1E12 with increasing amount of MUC1 (20AA). Molar ratios (binding site) are indicated for each spectrum.

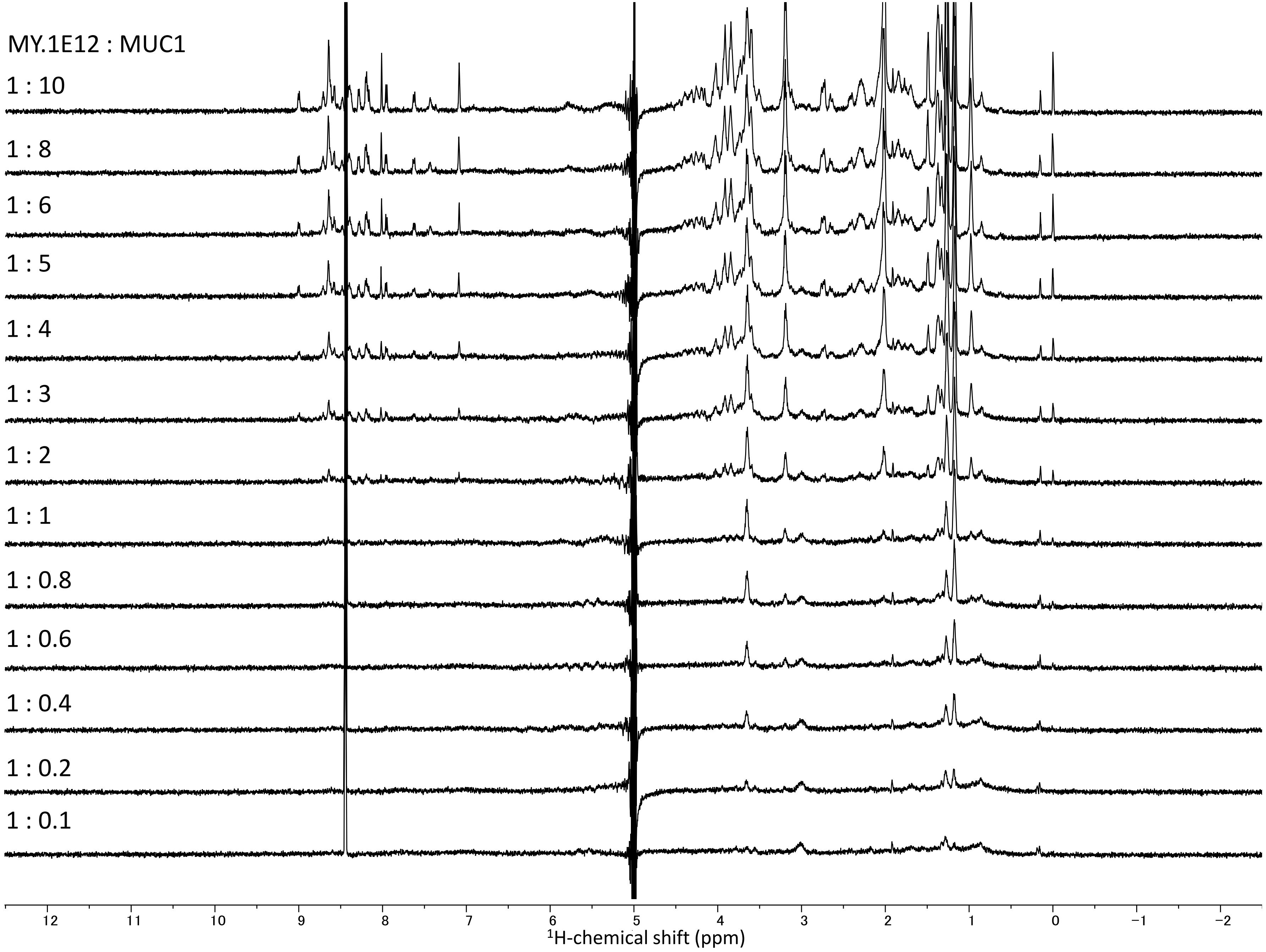

Figure S18. NMR titration experiments using MUC1 (20AA) and MY.1E12. 1D-1H NMR spectra (full region) of MY.1E12 with increasing amount of MUC1 (20AA). Molar ratios (binding site) are indicated for each spectrum.

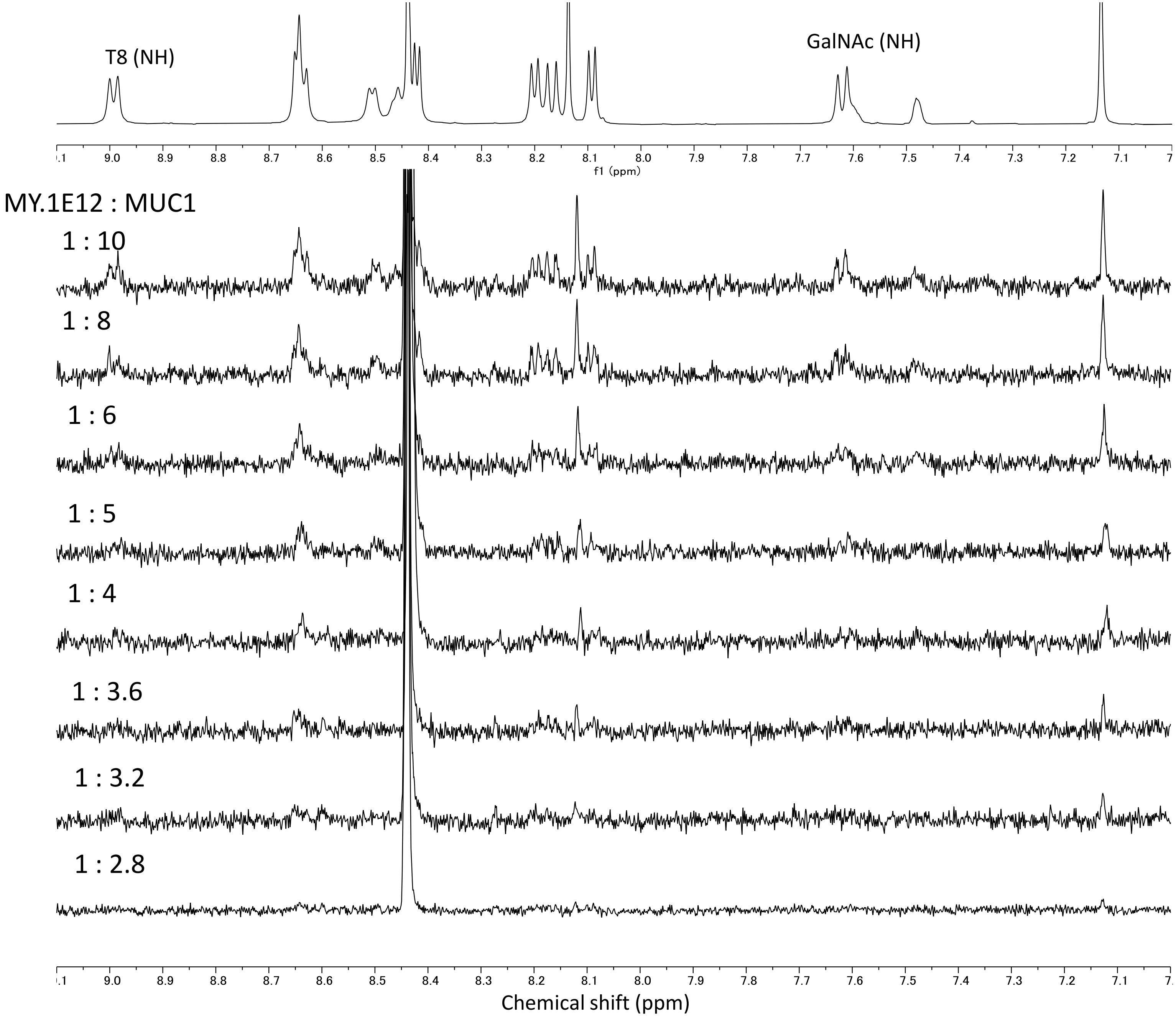

Figure S19. NMR titration experiments using MUC1 (9AA) and MY.1E12. 1D-^{1}H NMR spectra (NH region) of MY.1E12 with increasing amount of MUC1 (9AA). Molar ratios (binding site) are indicated for each spectrum.

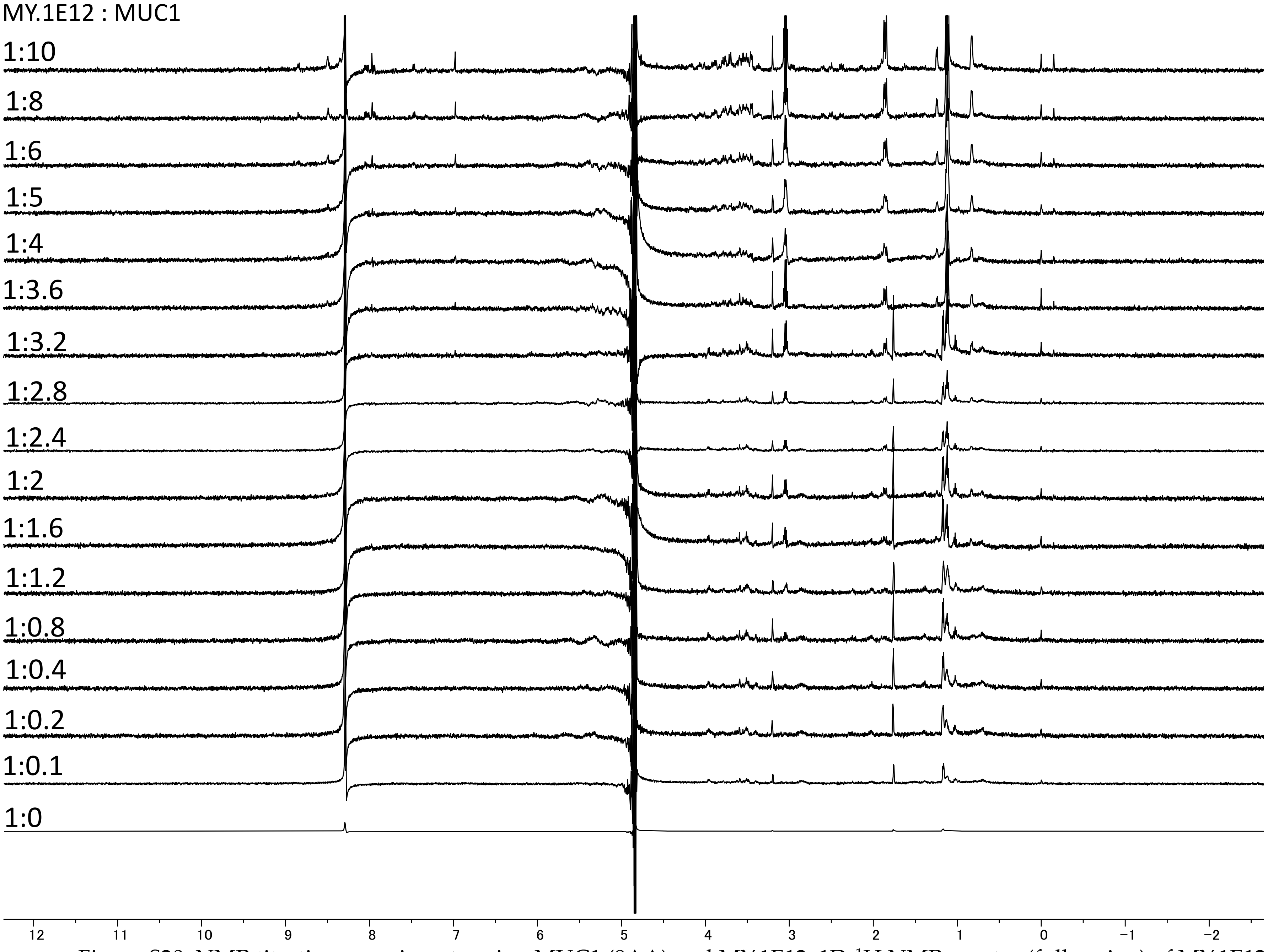

Figure S20. NMR titration experiments using MUC1 (9AA) and MY.1E12. 1D-^{1}H NMR spectra (full region) of MY.1E12 with increasing amount of MUC1 (9AA). Molar ratios (binding site) are indicated for each spectrum.

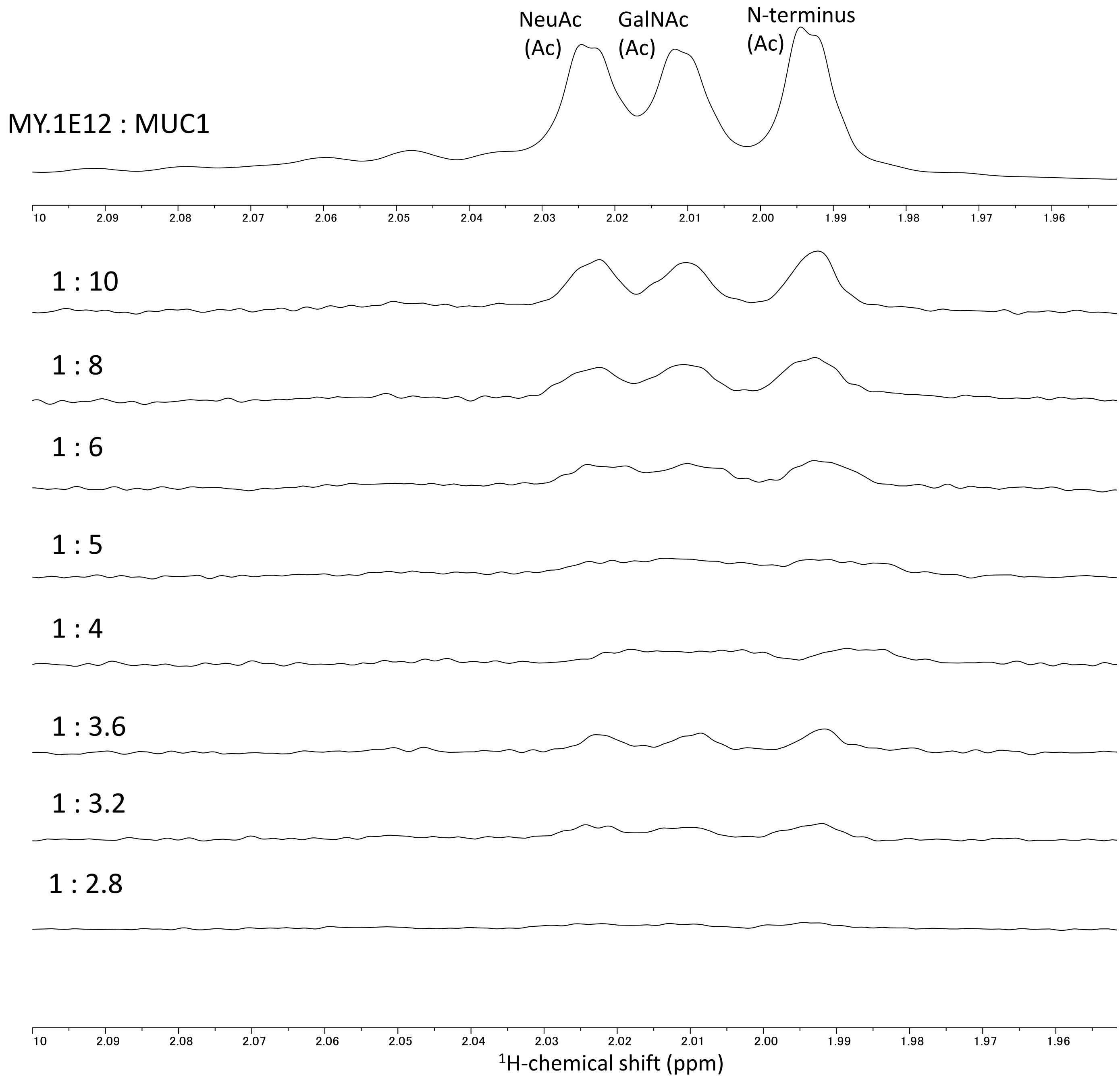

Figure S21. NMR titration experiments using MUC1 (9AA) and MY.1E12. 1D-^{1}H NMR spectra (acetyl region) of MY.1E12 with increasing amount of MUC1 (9AA). Molar ratios (binding site) are indicated for each spectrum.

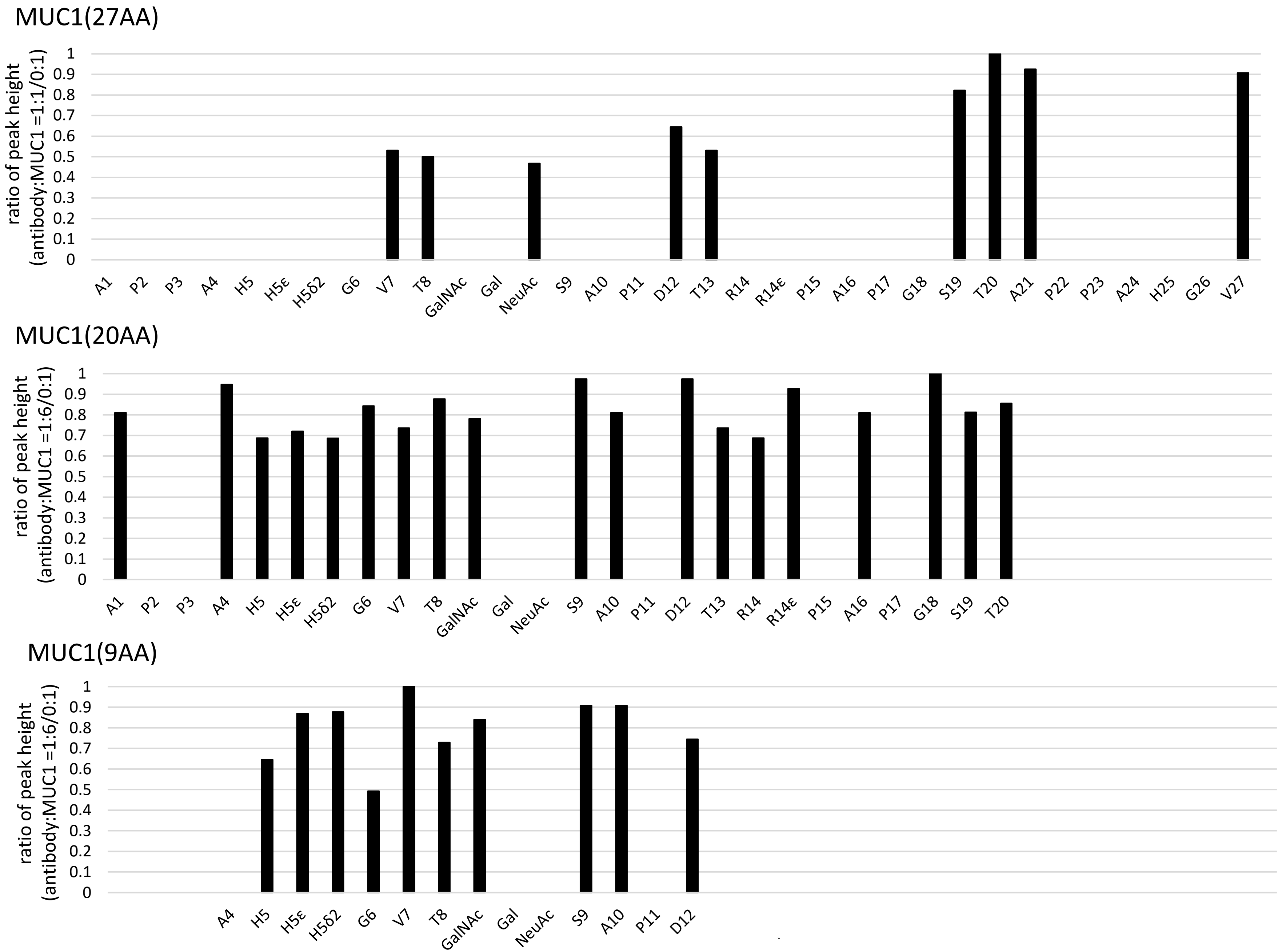

Figure S22. Ratio of peak height (MUC1+antibody/MUC1 alone) for each NH-Hα peak in CLIP-COSY spectra(27AA) and 1D-^{1}H NMR spectra(20AA, 9AA). The ratios are normalized against the respective highest value. All experiments were performed on a 600 MHz spectrometer at 278 K.

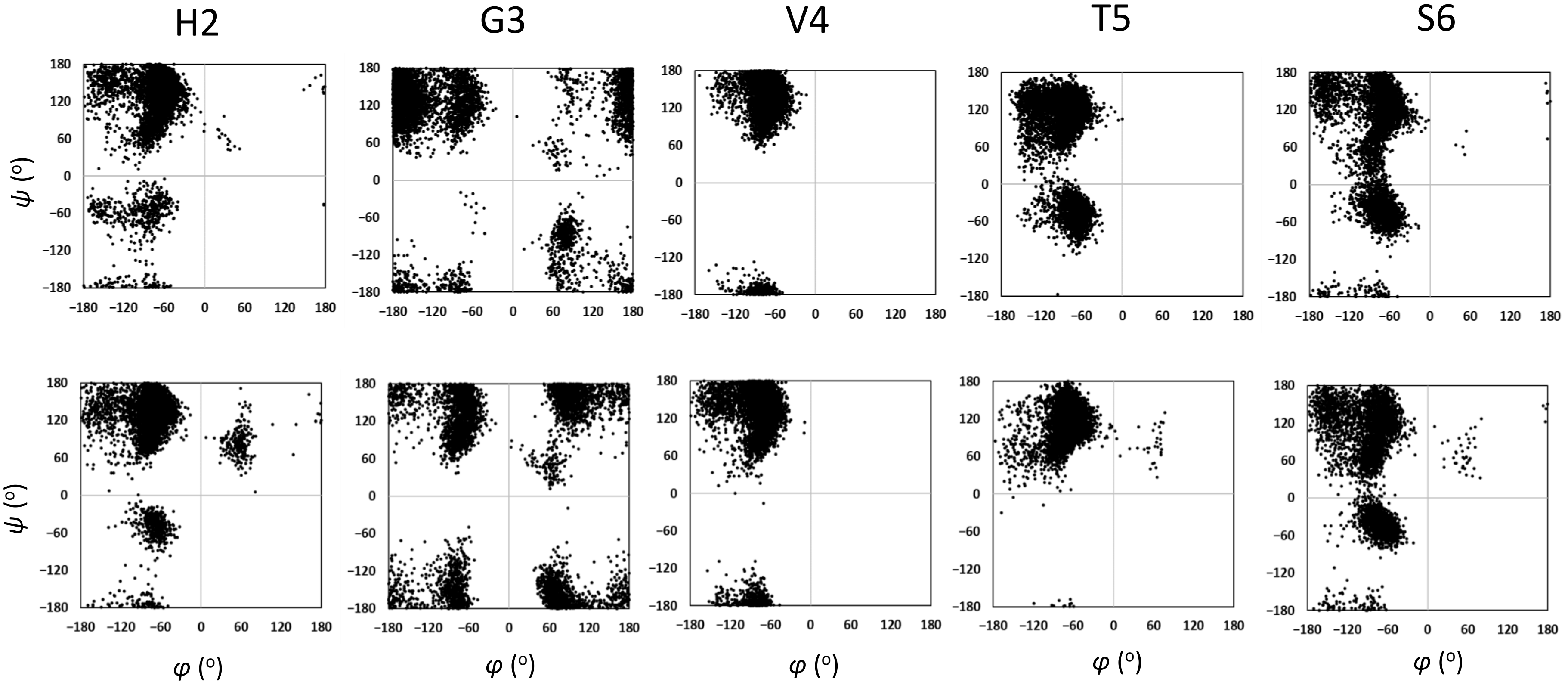

Figure S23. Distribution of dihedral angles of peptide constituting MUC1 (9AA) obtained by MD simulation. *O*-glycosylated MUC1 peptide (top) and unglycosylated peptide (below) were simulated.
